# Gratitude and problematic internet use in middle school students: the roles of perceived social support and self-harmony

**DOI:** 10.3389/fpsyg.2026.1937434

**Published:** 2026-10-14

**Authors:** Xiao Xiao, Qingyin Xiao, Yuting Liu, Youlong Zhan

**Affiliations:** 1 Department of Psychology, Hunan First Normal University, Changsha, China; 2 Department of Psychology, Hunan University of Science and Technology, Xiangtan, China

**Keywords:** gratitude, middle school students, perceived social support, problematic internet use, self-harmony

## Abstract

With the rapid development of the internet, its penetration among Chinese middle school students has neared saturation, and the issue of problematic internet use has become increasingly prominent. Therefore, the protective factors that curb this behavior warrant in-depth research. This study surveyed 1,255 middle school students to investigate the association between gratitude and problematic internet use as well as the potential mediating roles of perceived social support and self-harmony. The results indicated a significant negative correlation between gratitude and problematic internet use. Furthermore, gratitude was significantly associated with problematic internet use, and this association was mediated by perceived social support and self-harmony individually as well as through their serial mediation. These findings may inform future interventional research and theory development on problematic internet use.

## 1 Introduction

According to the ‘5th National Survey on Internet Usage Among Minors’, released in 2023 (CNNIC, 2023), the quantity of underage internet users in China exceeded 193 million, with a 99.20% penetration rate among middle school students, reaching saturation. It is noteworthy that 24.05% of underage internet users spend more than 2 h online every day, while 20.20% report feeling ‘very’ or ‘relatively’ dependent on the internet. Despite the implementation of targeted regulatory measures and educational guidance, these interventions have shown limited effectiveness: a substantial proportion of adolescents continue to exhibit problematic use, and comparisons between those who receive and do not receive such guidance reveal no consistent reduction in problematic internet use (Yang et al., 2023). Problematic internet use (PIU) refers to compulsive behavior characterized by excessive engagement with the internet, which exerts negative consequences on various aspects of an individual’s life (Beard and Wolf, 2001). PIU is typically operationalized through scores on validated scales; in the present study, adolescents scoring above established cut-off values on the Adolescent Pathological Internet Use Scale were classified as exhibiting PIU. Common online activities among middle school students include social networking, online gaming, short-video viewing, and information seeking, among which social networking and gaming are most strongly associated with PIU (Li et al., 2026). Assuming that ‘all middle school students are online’, the pressing need to address problematic internet use by identifying protective factors that can mitigate the risk of this behavior has become a cause of concern. The rise of positive psychology has led to growing academic interest in gratitude. Previous research on gratitude has predominantly focused on positive behaviors, while studies addressing negative behaviors remain relatively scarce. Using a questionnaire survey, this study investigated how gratitude is associated with problematic internet use, a prevalent negative behavior among middle school students, and the underlying mechanisms involving perceived social support and self-harmony.

### 1.1 Gratitude and problematic internet use

Gratitude is a traditional virtue in Chinese culture, exemplified by proverbs such as ‘A drop of water in grace should be repaid with a gushing spring’, that is passed down through generations. In psychology, gratitude is an individual’s disposition to cognitively recognize, affectively appreciate, and behaviorally reciprocate the support and assistance received from others, leading to positive experiences and outcomes (McCullough et al., 2002). It can be categorized into two levels: state gratitude, which is transient and context-dependent, and trait gratitude, which is stable and transcends specific situations (Froh et al., 2010). This study conceptualizes gratitude as a trait and examines its relationship with problematic internet use among middle school students. Trait gratitude plays a critical role in adapting individuals to the environment, such as helping them reduce addictive behaviors (Chen, 2017). At the theoretical level, the Broaden-and-Build Theory of Gratitude suggests that gratitude not only broadens an individual’s cognitive scope but also builds personal resources such as interpersonal support (Fredrickson, 2004). The Coping Hypothesis of Gratitude posits that greater dispositional gratitude fosters the adoption of more positive coping strategies (Wood et al., 2007). These theories from different perspectives demonstrate that gratitude possesses strong adaptive functions that reduce the risk of problematic behaviors. Empirically, previous studies have indicated that gratitude is a significant factor in mitigating problematic internet use. Specifically, individuals with stronger gratitude tendencies exhibit lower levels of pathological internet use (PIU), social media addiction, and internet gaming addiction (Yu et al., 2012; Gan et al., 2022; Ma et al., 2022; Cao et al., 2023; Wei, 2024). Furthermore, a longitudinal study demonstrated that individuals with higher gratitude levels were less likely to develop internet gaming addiction (Gao et al., 2023). In general, numerous theoretical models and research findings support the positive effect of gratitude on problematic internet use. This study proposes:

*H1*: In a sample of middle school students, gratitude negatively predicts problematic internet use.

### 1.2 Mediating role of perceived social support

Perceived social support denotes an individual’s conviction and assessment of the understanding, assistance, and support that they believe they can obtain from others (Wethington and Kessler, 1986; Wright and Wachs, 2023). Perceived social support serves as a critical protective factor in an individual’s environment and significantly influences their development (Yang et al., 2024). According to the Find-Remind-and-Bind Theory of Gratitude, gratitude may foster an increase in an individual’s perceived social support (Algoe, 2012; Algoe et al., 2013). On the one hand, gratitude helps individuals establish and consolidate positive social relationships, thereby increasing their access to external support (Kong et al., 2015). Relevant studies have found that individuals with a stronger disposition toward gratitude usually receive more social support (Luo and Mu, 2017; You et al., 2020; Chen et al., 2023). On the other hand, gratitude aids individuals in more acutely recognizing the understanding, support, and assistance offered by others, functioning as a ‘benefit detector’ (McCullough et al., 2008). Previous research demonstrated that gratitude enhances perceived social support (Wood et al., 2007; Giulia et al., 2020). Extensive research has confirmed that perceived social support is a significant negative predictor of both internet addiction and problematic internet use (Wu et al., 2016; Ye et al., 2017; Lei et al., 2018; Wang and Zhang, 2020). This suggests that problematic internet use may result from low levels of perceived social support, as proposed by relevant theoretical models. The Cognitive-Behavioral Model of PIU posits that lack of social support serves as a proximal trigger for PIU (Davis, 2001). The Compensatory Internet Use Theory explains that when psychological needs remain unmet in real life, some individuals turn to the internet for compensatory gratification (Kardefelt-Winther, 2014). Compared to non-addicted individuals, internet addicts tend to seek online connections and social support for their psychological needs (Ye et al., 2017). The Uses and Gratifications Theory points out that fulfilling psychological needs through the internet increases the frequency of an individual’s internet use and can even exacerbate the degree of immersion (Ko et al., 2005). In addition, some studies have discovered that gratitude can help individuals feel greater social support, which in turn inhibits their negative emotions and behaviors (Xiang et al., 2018; You et al., 2020). This study proposes:

*H2*: Among middle-school students, the relationship between gratitude and problematic internet use is mediated by perceived social support.

### 1.3 Mediating role of self-harmony

Self-harmony is one of the important indicators of mental health. It refers to a state characterized by the absence of salient conflicts within an individual’s self-concept and congruence between the self and experiences (Rogers, 1961). According to the Broaden-and-Build Theory of Gratitude, gratitude not only broadens the scope of cognition and builds personal resources but also promotes more positive appraisals of the self, others, and the external world (Fredrickson, 2004). Relevant studies indicate that individuals characterized by higher levels of gratitude are more likely to develop more positive self-concepts (Michailidis, 2021). These positive self-concepts and evaluations reduce internal conflicts and promote self-harmony. The Coping Hypothesis of Gratitude suggests that gratitude can motivate individuals to adopt more positive coping strategies, thus effectively dealing with internal conflicts (Wood et al., 2007), and contributing to the maintenance of self-harmony. Previous studies have found that individuals with stronger gratitude tendencies have a higher level of self-harmony (He et al., 2012; Chen et al., 2016). Moreover, while exploring self-identity, adolescents tend to experience role confusion due to internal conflicts and external pressures, making it difficult to achieve self-identity, that is, a state of self-harmony (Erikson, 1968). Studies have shown that individuals with lower self-harmony are more prone to smartphone addiction (Liu and Yao, 2016; Li et al., 2021). The Cognitive-Behavioral Model of PIU posits that maladaptive cognition is one of the key factors leading to an individual’s excessive reliance on the internet (Davis, 2001). Maladaptive cognition often manifests as negative self-concept and evaluation, which tends to emerge when an individual’s self-identity and psychological harmony are disrupted. In other words, when internal harmony is compromised, individuals may shift to online interaction as a coping mechanism to address the discomfort caused by internal conflicts. Furthermore, emotional factors such as shyness can exert influence on an individual’s internet addiction through self-harmony (Yu et al., 2019). This study proposes:

*H3*: In a sample of middle school students, self-harmony mediates the connection between gratitude and problematic internet use.

### 1.4 Chain-mediating role of perceived social support and self-harmony

According to the Main Effect Model and the Stress-Buffering Model, perceived strong social support networks not only enhance an individual’s self-esteem and self-confidence while promoting physical and mental health, but also mitigate an individual’s perception of threat from stressors, thereby protecting the self-concept from being undermined (Cobb, 1976; House et al., 1988). During self-identity development, individuals who perceive greater social support are more inclined to actively explore and have a stronger willingness to make commitments. This enables them to clearly define their role positioning across different environments, thereby gradually forming a more harmonious and consistent self-concept (Kroger, 2002). Previous research has established that social support positively predicts self-harmony (Gu et al., 2016). Favorable interpersonal relationships are strongly correlated with elevated levels of self-harmony (Ye and Ye, 2020). Chen et al. (2016) identified the mediating role of perceived social support in the relationship between gratitude and self-harmony. This finding implies that gratitude may indirectly promote self-harmony by strengthening perceived social support. This study proposes:

*H4*: Among middle school students, perceived social support and self-harmony sequentially mediate the relationship between gratitude and problematic internet use.

In summary, gratitude, a positive emotional trait, influences problematic internet use through multiple pathways according to existing theoretical models and research findings. This study aimed to investigate the relationship between gratitude and problematic internet use among middle school students, and the roles played by perceived social support and self-harmony. Figure 1 illustrates the hypothesized chain mediation model examined in this study.

**Figure 1:**
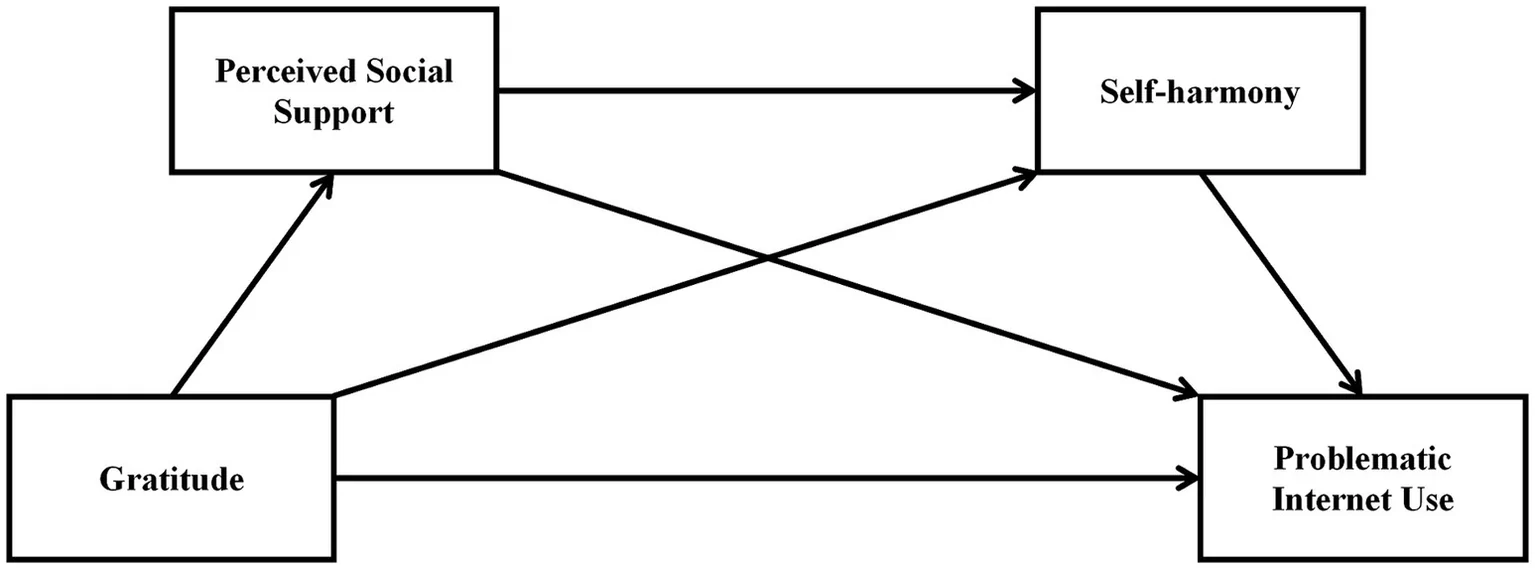
Proposed chain mediation model.

## 2 Methods

### 2.1 Participants

This study employed a cluster sampling method. Three middle schools were conveniently selected in Hunan and Guangdong Provinces, China, on the basis of their accessibility, geographic diversity across the two provinces, and willingness to participate. Among these schools, one was located in an urban setting and two in suburban or rural settings, thereby enhancing the representativeness of the sample. All classes across all grade levels within these schools constituted the sampling frame, from which a number of classes were randomly selected (cluster sampling conducted at the class level). All students in the ultimately chosen classes were surveyed. The inclusion criteria were: (1) current enrolment as a middle school student; and (2) provision of informed consent (from legal guardians) and assent (from participants). Students with severe cognitive or psychiatric conditions that would impair questionnaire completion were excluded. *A priori* power analysis using G*Power 3.1.9.7 (Faul et al., 2007, 2009) was conducted to determine the minimum required sample size. Because a serial mediation model (PROCESS Model 6) is estimated through a series of ordinary least squares regression equations, we based the calculation on the regression equation containing the largest number of predictors (six predictors), assuming a medium effect size (*f* ^2^ = 0.15), *α* = 0.05, and power = 0.80, which indicated that a minimum sample of 98 participants was required; our final sample of 1,255 participants substantially exceeded this requirement. Questionnaires with excessive missing data or patterned responses would also be excluded from analysis.

### 2.2 Measures

#### 2.2.1 Gratitude

This study used the Gratitude Questionnaire-6 (GQ-6) to assess trait gratitude (McCullough et al., 2002; Wei et al., 2011). The GQ-6 has been validated in Chinese-speaking adolescents and demonstrates a unidimensional factor structure. The scale consisted of 6 items (sample item: ‘I want to thank all kinds of people’) rated on a 7-point Likert scale ranging from ‘strongly disagree’ to ‘strongly agree’. Two items were reverse-scored. Higher scores indicated a stronger dispositional tendency toward gratitude. The internal consistency in this study was adequate (*α* = 0.79).

#### 2.2.2 Perceived social support

This study employed the Multidimensional Scale of Perceived Social Support (MSPSS) to assess perceived social support (Zimet et al., 1988; Jiang, 2011). The MSPSS has been validated in Chinese-speaking adolescents and demonstrates a three-factor structure. The scale consisted of 12 items, which were divided into three dimensions: perceived family support (sample item: ‘My family can provide concrete help to me’), perceived friend support (sample item: ‘My friends can genuinely help me’), and perceived other support (sample item: ‘I can share joys and sorrows with certain people such as teachers, classmates, relatives’). Items were rated on a 7-point Likert scale ranging from ‘very strongly disagree’ to ‘very strongly agree’, with no reverse-scored items. Higher scores indicated a higher level of perceived social support. The internal consistency in this study was excellent (*α* = 0.91).

#### 2.2.3 Self-harmony

This study utilized the Self-Consistency and Congruence Scale (SCCS) to assess self-harmony (Rogers, 1961; Wang, 1994). The SCCS has been validated in Chinese-speaking adolescents and demonstrates a three-factor structure. The scale consisted of 35 items, which were divided into three dimensions: self-experience incongruence (sample item: ‘People around me often think my self-perception is somewhat contradictory’), self-flexibility (sample item: ‘I feel that many things I have done should not have been done’), and self-rigidity (sample item: ‘Some of my friends are very different from me, but this does not affect our relationship’). Items were rated on a 5-point Likert scale, ranging from ‘completely inconsistent’ to ‘completely consistent’, with several items reverse-scored. According to the original SCCS development study (Wang, 1994), higher scores on the Self-Experience Incongruence and Self-Rigidity subscales indicate lower levels of self-harmony, whereas higher scores on the Self-Flexibility subscale indicate higher levels of self-harmony. To ensure that higher total scores uniformly reflect greater self-harmony, we reverse-scored the Self-Experience Incongruence and Self-Rigidity subscales before computing the total SCCS score. Consequently, in the present study, higher total SCCS scores indicate higher levels of self-harmony. The internal consistency in this study was acceptable (*α* = 0.77).

#### 2.2.4 Problematic internet use

This study employed the Adolescent Pathological Internet Use Scale to assess problematic internet use (Lei and Yang, 2007). The scale has been validated in Chinese-speaking adolescents and demonstrates a six-factor structure. The scale consisted of 38 items categorized into six dimensions: salience (sample item: ‘Once I go online, I do not think about anything else’), tolerance (sample item: ‘Compared to before, I need to spend more time online to feel satisfied’), withdrawal reaction (sample item: ‘When unable to access the internet, I feel intensely curious about what is happening online’), mood alteration (sample item: ‘When I encounter troubles, using the internet cheers me up a bit’), social problems (sample item: ‘I feel more secure when interacting with others online’), and negative consequences (sample item: ‘internet use has negatively impacted my physical health’). Items were rated on a 5-point Likert scale, ranging from ‘completely inconsistent’ to ‘completely consistent’, with no reverse-scored items. Higher scores indicated a stronger tendency toward problematic internet use. The internal consistency in this study was excellent (*α* = 0.95).

#### 2.2.5 Family socioeconomic status

Family socioeconomic status (SES) was measured by a single self-report item assessing subjective family economic conditions, rated on a 5-point Likert scale ranging from 1 (very poor) to 5 (very good). Higher scores indicate better perceived family economic conditions.

### 2.3 Procedures

Written informed consent was obtained from legal guardians, and assent was obtained from the participants themselves, prior to the commencement of the study procedures. After reading and understanding the instructions, the participants completed all paper questionnaires in a quiet classroom, which took approximately 25 min. Participants were informed that participation was voluntary and that they could decline to participate or withdraw at any time without consequence. Questionnaires were completed individually, and to ensure confidentiality, responses were collected anonymously and immediately placed in sealed envelopes by the participants themselves. No teachers were present in the classroom during questionnaire completion, in order to minimize any influence on participant responses. Trained research assistants were available throughout the session to answer questions and to provide support in the event that any participant became distressed by the questions.

### 2.4 Data statistics and analysis

All statistical analyses were conducted using SPSS 24.0. First, descriptive statistics and Pearson correlations among the study variables were computed. Second, the chain mediation model was tested using Model 6 in the PROCESS macro of SPSS (Hayes, 2013), with 5,000 bootstrap resamples and 95% bias-corrected confidence intervals. Gratitude was specified as the predictor, problematic internet use as the outcome, and perceived social support and self-harmony as sequential mediators. Family socioeconomic status, age, and gender were included as covariates; family socioeconomic status was selected because it showed significant correlations with the study variables, while age and gender were included given the wide age range (12–18 years) and balanced gender composition of the sample. Finally, common method bias was assessed using Harman’s single-factor test and a confirmatory factor analysis with a single latent factor, because the self-reported nature of the data could inflate observed associations. To account for the cluster sampling at the class level, we computed the intraclass correlation coefficient (ICC = 0.03) and applied cluster-robust standard errors at the class level in the mediation analysis to correct for potential bias in standard errors and hypothesis testing.

## 3 Results

### 3.1 Descriptive statistics and correlations

Of the 1,340 questionnaires distributed, 1,255 valid responses were obtained (effective rate: 93.70%). The 85 invalid responses were excluded because of excessive missing data, patterned (straight-line) responses, or failure to meet the inclusion criteria. Descriptive statistics are presented in Table 1. The correlation analysis (see Table 1) indicated that gratitude was significantly positively correlated with perceived social support and self-harmony, while it was significantly negatively correlated with problematic internet use. A significant positive correlation was found between perceived social support and self-harmony, whereas both variables were significantly negatively correlated with problematic internet use. Additionally, family socioeconomic status showed significantly negative correlations with gratitude, perceived social support, and self-harmony. Although this negative association may appear counterintuitive, it may reflect the higher academic and parental expectations faced by adolescents from higher-SES families, and it should be interpreted with caution given that the measure reflects subjective rather than objective family economic conditions. Family socioeconomic status was therefore controlled for in the subsequent mediation analysis. Regarding the demographic composition of the sample, among the 1,255 participants, 665 (52.99%) were female and 590 (47.01%) were male; 647 (51.55%) were junior high school students and 608 (48.45%) were senior high school students. Participants’ ages ranged from 12 to 18 years, with a mean age of 14.89 years (SD = 1.77).

**Table 1:** Descriptive statistics and bivariate correlations between variables.

Variable	M	SD	1	2	3	4	5	6	7
1. Sex	—	—	1						
2. Age	14.89	1.77	0.04	1					
3. Family SES	2.89	0.64	0.05	0.12^**^	1				
4. Gratitude	4.87	1.10	0.01	−0.14^**^	−0.09^**^	1			
5. Perceived social support	4.82	1.08	0.01	−0.08^**^	−0.20^**^	0.54^**^	1		
6. Self-harmony	3.27	0.39	−0.06*	−0.09^**^	−0.16^**^	0.36^**^	0.50^**^	1	
7. Problematic internet use	2.42	0.72	−0.04	0.01	0.07*	−0.28^**^	−0.31^**^	−0.39^**^	1

*N* = 1,255. For sex, 665 participants (52.99%) were female and 590 (47.01%) were male; frequencies and percentages are reported in the text (Section 3.1). ^*^p < 0.05, ^**^p < 0.01, ^***^p < 0.001.

### 3.2 Common method bias test

The self-reported nature of the data collection makes common method bias (CMB) a potential concern. Procedural remedies have been implemented to mitigate this issue, including anonymous responses and reverse-scored items. Moreover, Harman’s single-factor test indicated 26 factors with eigenvalues above 1, with the first factor explaining only 17.15% of the variance, which is well below the 40% threshold. To supplement this test, a confirmatory factor analysis in which all items were loaded onto a single latent factor yielded a poor model fit (CFI < 0.90, RMSEA > 0.10), further indicating that a single common factor did not account for the majority of the variance. Therefore, CMB was not a significant concern in this study.

### 3.3 Testing for chain mediation of perceived social support and self-harmony

Based on the correlation results, a serial mediation analysis was conducted using Model 6 of the PROCESS macro to test the hypothesized chain of mediation. The model specified gratitude as the predictor, problematic internet use as the outcome, and perceived social support and self-harmony as sequential mediators, while controlling for the influence of family socioeconomic status, age, and gender.

Regression analysis (see Table 2) indicated that gratitude was a significant negative predictor of problematic internet use (*β* = −0.28, *p* < 0.001), and a positive predictor of both perceived social support (*β* = 0.53, *p* < 0.001) and self-harmony (*β* = 0.13, *p* < 0.001). Perceived social support was a significant positive predictor of self-harmony (*β* = 0.42, *p* < 0.001). In a combined model, gratitude (*β* = −0.12, *p* < 0.001), perceived social support (*β* = −0.10, *p* < 0.01), and self-harmony (*β* = −0.30, *p* < 0.001) significantly and negatively predicted problematic internet use.

**Table 2:** Regression analysis of variable relationships in the chain mediation model.

Result variable	Predictor variables	*R*	*R^2^*	*F*	*β*	*t*
Problematic internet use	Gratitude	0.28	0.08	36.15^***^	−0.28	−10.10^***^
	Family socioeconomic status				0.08	1.78
	Age				−0.03	−1.25
	Sex				−0.04	−1.40
Perceived social support	Gratitude	0.56	0.32	193.18^***^	0.53	22.46^***^
	Family socioeconomic status				−0.24	−6.42^***^
	Age				0.01	0.61
	Sex				0.01	0.28
Self-harmony	Gratitude	0.52	0.27	113.23^***^	0.13	4.31 ^***^
	Perceived social support				0.42	14.21^***^
	Family socioeconomic status				−0.10	−2.60^**^
	Age				−0.03	−1.07
	Sex				−0.06	−2.46*
Problematic internet use	Gratitude	0.43	0.18	55.66^***^	−0.12	−3.86^***^
	Perceived social support				−0.10	−2.98 ^**^
	Self-harmony				−0.30	−10.16^***^
	Family socioeconomic status				−0.01	−0.19
	Age				−0.04	−1.51
	Sex				−0.05	−2.14*

^*^p < 0.05, ^**^p < 0.01, ^***^p < 0.001.

The results of the mediation effect analysis (see Table 3 and Figure 2) show that the effect of gratitude on problematic internet use was characterized by a partial mediation model. The total effect was −0.28 (SE = 0.03, *t* = −10.10, *p* < 0.001, 95% CI = [−0.33, −0.22]). The direct effect of gratitude on problematic internet use was −0.12 (SE = 0.03, *t* = −3.86, *p* < 0.001, 95% CI = [−0.18, −0.06]), accounting for 42.86% of the total effect. The total mediating effect was −0.16 (SE = 0.02, 95% CI = [−0.20, −0.12]), accounting for 57.14% of the total effect. The specific mediating pathways include: (1) Gratitude → Perceived Social Support → Problematic Internet Use (effect value = −0.05, SE = 0.02, 95% CI = [−0.09, −0.02], accounting for 17.86% of the total effect); (2) Gratitude → Self-harmony → Problematic Internet Use (effect value = −0.04, SE = 0.01, 95% CI = [−0.06, −0.02], accounting for 14.29% of the total effect); (3) Gratitude → Perceived Social Support → Self-harmony → Problematic Internet Use (effect value = −0.07, SE = 0.01, 95% CI = [−0.09, −0.05], accounting for 25.00% of the total effect). The confidence intervals for all pathways did not contain zero, indicating a significant mediating effect.

**Table 3:** Mediation effect test of the chain mediation model.

Effect	Path	Effect size	SE	LLCI	ULCI	Proportion of effect size (%)
Total effect	Gratitude → Problematic internet use	−0.28	0.03	−0.33	−0.22	100
Direct effect	Gratitude → Problematic internet use	−0.12	0.03	−0.18	−0.06	42.86
Total mediating effect		−0.16	0.02	−0.20	−0.12	57.14
Mediating effect 1	Gratitude → Perceived social support → Problematic internet use	−0.05	0.02	−0.09	−0.02	17.86
Mediating effect 2	Gratitude → Self-harmony → Problematic internet use	−0.04	0.01	−0.06	−0.02	14.29
Mediating effect 3	Gratitude → Perceived social support → Self-harmony → Problematic internet use	−0.07	0.01	−0.09	−0.05	25.00

**Figure 2:**
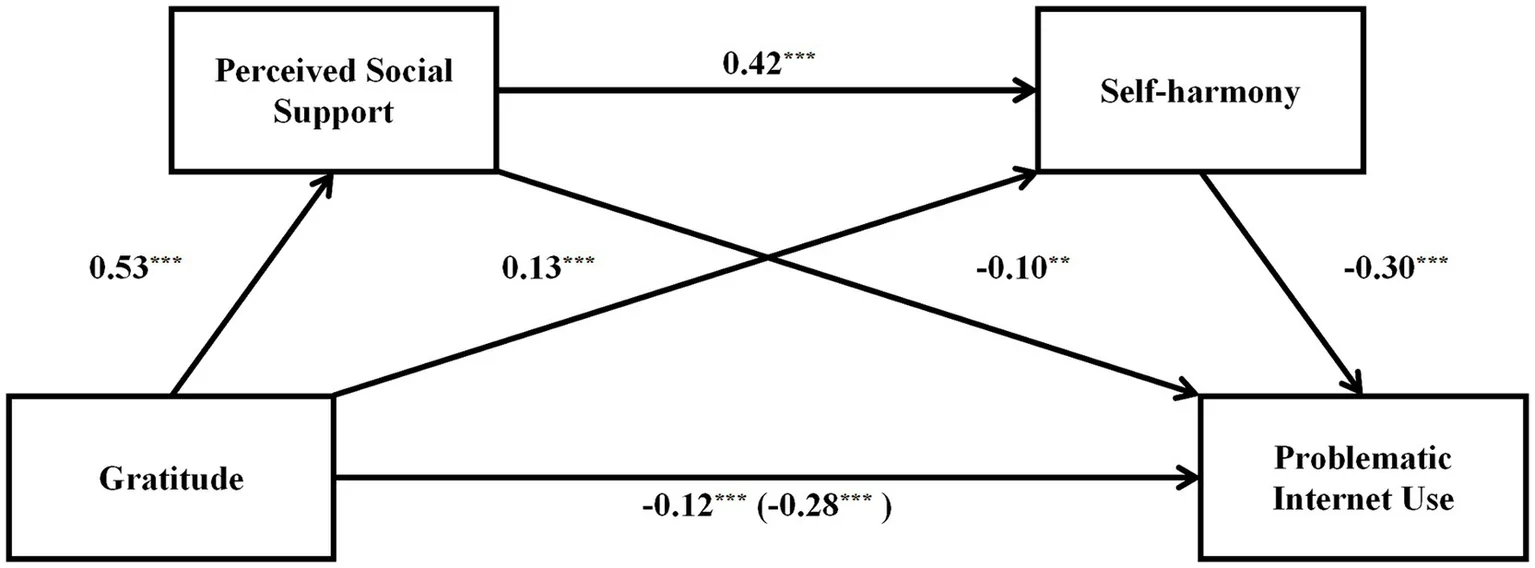
Chain mediation model. ^*^p < 0.05, ^**^p < 0.01, ^***^p < 0.001.

## 4 Discussion

### 4.1 Gratitude and problematic internet use

It was found that gratitude negatively predicted problematic internet use, which supported Hypothesis 1. Consistent with previous research results, the lower the level of gratitude, the more serious the problematic internet use of individuals (Yu et al., 2012; Ma et al., 2022; Cao et al., 2023), which indicates that gratitude is associated with lower problematic internet use among middle school students. Simultaneously, the findings of this study are consistent with the Broaden-and-Build Theory of Gratitude and the Coping Hypothesis of Gratitude. Specifically, gratitude broadens cognitive scope and builds personal resources, thereby enhancing an individual’s adaptability to his/her environment. This enhanced adaptability may enable individuals to develop greater resistance to online temptations (Fredrickson, 2004; Yu et al., 2012). Furthermore, people with stronger gratitude tendencies typically focus more on the positive aspects of life. When facing stress or distress, they actively adopt constructive coping strategies rather than resorting to internet use as a means of escape (Wood et al., 2007; Yu et al., 2012). Therefore, gratitude may help individuals maintain rationality in complex online environments and avoid problematic internet use.

### 4.2 Mediation of perceived social support

The supported mediating role of perceived social support between gratitude and problematic internet use provides empirical support for Hypothesis 2. This suggests that gratitude is associated with lower problematic internet use through higher perceived social support. Specifically, people with gratitude are not only more likely to acquire social support but also more adept at detecting the understanding, support, and assistance provided by others (Algoe, 2012; Algoe et al., 2013). Consequently, the more inclined one is toward gratitude, the greater one’s perceived level of social support (Wood et al., 2007; Giulia et al., 2020). Notably, if the need for social support could be sufficiently met through real-life relationships, his/her intrinsic motivation to seek compensatory satisfaction in a virtual space would be reduced. This is consistent with the Cognitive-Behavioral Model of PIU and the Compensatory Internet Use Theory. When people lack sufficient social support in offline life, the internet readily becomes an alternative channel for fulfilling psychological needs, thereby increasing the risk of problematic internet use (Davis, 2001; Kardefelt-Winther, 2014). Thus, cultivating gratitude in middle school students may have important practical implications. It may be associated with a greater capacity to perceive social support and serve as a protective factor against problematic internet use.

### 4.3 Mediation of self-harmony

The significant mediating effect of self-harmony supported Hypothesis 3, demonstrating its role in the link between gratitude and reduced problematic internet use. This finding is consistent with the interpretation that gratitude is associated with higher self-harmony, which in turn is associated with a lower risk of problematic internet use. Individuals with a stronger tendency toward gratitude typically exhibited higher levels of self-harmony, consistent with previous research findings (He et al., 2012; Chen et al., 2016). This may enable them to avoid discomfort arising from conflicts between themselves and their experiences. Consequently, it may help maintain mental health and reduce the likelihood of relying on social networks as a coping mechanism. In accordance with the Broaden-and-Build Theory of Gratitude and the Coping Hypothesis of Gratitude, gratitude fosters positive evaluations of the self, others, and the world on the one hand, while also helping alleviate negative emotions triggered by internal and external pressures on the other (Fredrickson, 2004; Wood et al., 2007). These two mechanisms may work synergistically to maintain and enhance self-harmony. In addition, individuals with greater self-harmony demonstrate a heightened capacity to accept their experiences and integrate their self-concepts, thereby mitigating maladaptive cognition stemming from unsuccessful identity exploration. This may further prevent them from seeking false self-identities through the internet. Therefore, by enhancing their level of self-harmony, gratitude may provide middle school students with an intrinsic psychological protective mechanism so that they can remain rational and restrained in the process of internet use.

### 4.4 Chain mediating role of perceived social support and self-harmony

Hypothesis 4 was supported as perceived social support and self-harmony were identified as significant serial mediators in the relationship between gratitude and problematic internet use. In line with prior research, perceived social support was closely linked to self-harmony (Gu et al., 2016). A higher level of perceived social support helps individuals integrate self-concepts more effectively, reduces internal conflicts, and facilitates self-harmony. Simultaneously, the findings of this study are consistent with both the Main Effect Model and the Stress-Buffering Model. Perceived social support not only enhances an individual’s self-esteem and self-confidence but also provides the psychological resources necessary to cope with stress, thereby helping to maintain self-harmony more effectively when facing internal conflicts (Cobb, 1976; House et al., 1988). Consequently, gratitude may strengthen an individual’s perception of social support, laying a solid foundation to promote self-harmony. An enhanced level of self-harmony is further associated with a reduced risk of developing internet dependency. This pattern of results is consistent with a chain mediation process in which gratitude, a positive emotional trait, is related to problematic internet use through perceived social support and self-harmony, and offers new perspectives for developing preventive and intervention strategies targeting middle school students.

### 4.5 Limitations and future orientation

This study has some limitations. First, its cross-sectional design precludes causal inferences and prevents the establishment of temporal ordering among the variables in the mediation model; the findings should therefore be interpreted as consistent with the hypothesized pathway rather than as direct causal evidence. Given that adolescents are undergoing a period of rapid developmental change, future studies should employ longitudinal designs to track the dynamic evolution of these variables and elucidate their underlying causal mechanisms. Second, the assessment of common method bias relied primarily on Harman’s single-factor test, which, although supplemented by a confirmatory factor analysis, provides only indirect evidence; future studies could adopt more robust approaches such as the unmeasured latent method factor technique. Furthermore, this study identified several protective factors that may help to prevent and intervene in problematic internet use. However, the practical effectiveness of the measures associated with these factors in educational settings has not yet been fully validated, and trait gratitude, social support, and self-harmony may not be readily modifiable in typical educational contexts. Future research should conduct randomized controlled trials to further examine the efficacy of related measures in addressing problematic internet use. Lastly, gratitude is closely associated with numerous positive psychological traits and exerts a positive influence on individuals’ physical and psychological development. These factors are associated with reduced problematic internet use. Future research could build on this study to explore the interactions among other variables, thereby providing multiple theoretical pathways for addressing problematic internet use.

## 5 Conclusions and educational implications

A significant negative correlation was observed between gratitude and problematic internet use among middle school students. Furthermore, gratitude demonstrated significant direct and indirect associations with problematic internet use. Specifically, the indirect associations operated through three pathways: the simple mediation of perceived social support, the simple mediation of self-harmony, and the serial mediation of perceived social support and self-harmony. These findings highlight the potential value of gratitude in relation to the prevention and alleviation of problematic internet use among adolescents, and may inform future interventional research.

Based on the observed associations in this study, the following three approaches are tentatively recommended as potential directions for future intervention research, noting that their efficacy requires empirical validation and that trait gratitude, social support, and self-harmony may not be readily modifiable in typical educational settings. First, educators could guide students to record, express, and reflect on gratitude, thereby promoting communication with those around them and fulfilling their emotional needs in real-life contexts. Second, educators could encourage students to interpret others’ intentions and behaviors positively, enhancing their ability to perceive, recognize, and utilize understanding, support, and assistance from people in their environment. Third, educators could assist students in developing accurate self-awareness and self-evaluation, helping to resolve conflicts between their self-concepts and experiences to achieve self-harmony.

## Data Availability

The datasets presented in this study can be found here: https://osf.io/yrmd8/overview?view_only=8001b7a7f5e94e66ba4bea8c7736ceba. Further inquiries can directed to the corresponding author/s.
